# Editorial: Microbial ecological and biogeochemical processes in the soil-vadose zone-groundwater habitats, volume II

**DOI:** 10.3389/fmicb.2024.1486331

**Published:** 2024-09-09

**Authors:** Huai Li, Zifang Chi, Jiuling Li, Yihao Luo

**Affiliations:** ^1^State Key Laboratory of Black Soils Conservation and Utilization, Northeast Institute of Geography and Agroecology, Chinese Academy of Sciences, Changchun, China; ^2^Key Lab of Groundwater Resources and Environment, Ministry of Education, Jilin University, Changchun, China; ^3^Australian Centre for Water and Environmental Biotechnology, The University of Queensland, Brisbane, QLD, Australia; ^4^Swette Center for Environmental Biotechnology, Biodesign Institute at Arizona State University, Tempe, AZ, United States

**Keywords:** soil, vadose zone, groundwater, habitats, microbial ecological and biogeochemical processes

Microorganisms regulate biogeochemical cycles and serve various functions within the soil, vadose zone, and groundwater habitats (Chi et al., 2022; Li et al., 2023). Microbial communities are highly sensitive to environmental changes and can respond rapidly to such alternations (Liang et al., 2023). The composition and functionality of microorganisms across different habitats are influenced by both biotic and abiotic factors, which in turn affect biochemical processes and overall ecosystem functions (Li et al., 2019, 2022). Natural wetlands, landfills, composts, vadose zones, and saturated aquifers are examples of the habitats typically found within soil, vadose zone, and groundwater. This topic aims to compile recent research on microbial ecological processes within the soil-vadose zone-groundwater continuum and to highlight the potential for achieving sustainable processes. There is significant interest in understanding these interconnected habitats, particularly regarding microbial pathways involved in material cycling, pollution control, and carbon neutrality. Thirteen articles included in this Research Topic have undergone rigorous peer review and have been selected for their contributions to these areas of study.

Ding et al. investigated the impact of key microorganisms on water quality stability within river-lake systems during periods of hydrological regulation. Their findings revealed that lake areas exhibited better water quality compared to both inflow and inflow areas, although no significant differences were observed in sediment composition. The study identified *Pseudomonas, Acinetobacter*, and *Microbacterium* as crucial in removal of nitrate and phosphates. However, an increase in flow velocity and nutrient load was found to negatively affect the abundance of these key microorganisms.

Li, Zhu et al. conducted short-term field soil incubation study using samples from the Svalbard glacier meltwater river at varying temperatures (2°C, 10°C, 20°C). The study found that CH_4_ emissions in soil warming did not increase in the first several days, but site specificity was more important. However, emissions initially increased before gradually decreasing as the warming period extended. These results are significant for assessing GHG emission fluxes under global warming.

Xu et al. isolated a bacterium named NS-6 from sandstone oil in the Ordos Basin. This strain demonstrated exceptional urease production and calcium carbonates (CaCO_3_) precipitation capabilities. Additionally, it was found to possess a serial of genes involved in urea catabolism and CaCO_3_ deposition.

Yuan et al. discovered that phosphorus (P) components significantly influence bacterial and archaeal β-diversity in sediments. In Hongfeng Lake, β-diversity was influenced by metal oxide-bound inorganic P and sediment total P, whereas in Aha Lake, it's affected by reductant-soluble total P or calcium-bound inorganic P. Inorganic P had greater effects on bacterial β-diversity, while organic P more affected archaeal diversity.

Wang et al. investigated the effects of various fertilization treatments on soil antibiotic resistance genes (ARGs) and found that organic fertilizers led to a higher number and abundance of ARGs. In contrast, the changes in ARGs associated with chemical fertilizers were primarily due to the colonization of native microorganisms, and conventional fertilizers were in between. This finding offers valuable insights into the dynamics of ARGs under long-term application of different fertilizers.

Li H. et al. investigated the effects of different depths of groundwater irrigation on soil microorganisms in paddy wetlands. Their study revealed that microorganisms in shallow groundwater irrigation was highly sensitive to environmental changes, and Fe-anammox, nitrification, and methane oxidation were favorable under deep groundwater irrigation. The findings offer new ideas for controlling non-point source pollution and reducing greenhouse gas emissions in paddy wetlands.

Zhang L. et al. studied the seasonal variation of nitrogen-cycling genes in the sediment of Baiyangdian and identified dissimilatory nitrate reduction, assimilatory nitrate reduction, and denitrification were dominant nitrogen-cycling processes. Nitrification-related genes had high abundance in spring, while high denitrification-related genes in fall were observed. Dissolved organic carbon, water temperature, and antibiotics were significantly correlated with nitrogen-cycling processes.

Zhang R. et al. evaluated the impact of water management on soil physicochemical properties and enzyme activities in greenhouse grape cultivation. They found that while water stress had a minimal effect on soil physical–chemical properties, it significantly reduced the accumulation of soil microbial biomass carbon (MBC) content throughout the grape growing season and reduced soil microbial biomass nitrogen (MBN) content in later growth. Mild and moderate water stress conditions were also found to inhibit the activities of urease, catalase, and sucrase activities.

Du et al. developed a water quality index (WQI) model to evaluate the water quality on Hainan Island using data from 2015 to 2020. The results indicated a moderate overall water quality, with 86.36% of monitoring stations classified as having good quality, while 13.53% were categorized as poor or very poor. Poor quality was primarily observed in major cities and aquaculture areas, with worst conditions in March, October, and November. Key pollution sources included urbanization, agriculture, and industry.

Li, Hong et al. investigated the effects of *Pythium ultimum* on the migration and biodegradation of bacteria Diaphorobacter sp. LW2 in soils of different particle sizes. They found that the hyphae of *Pythium ultimum* facilitated the growth and migration of LW2, enabling it to move along or against the direction of hyphae growth. Pythium ultimum enhanced the migration and survival of LW2 in soil, improving the bioremediation of polluted soil.

Song et al. reviewed the critical role of freshwater wetland biodiversity in maintaining habitat functional stability. They examined the environmental drivers affecting habitat function stability, explored the effects of plant and microbial diversity on habitat function stability, revealed the impacts and mechanisms of habitat changes on biodiversity, and further proposed an outlook for research in freshwater wetland research.

Ali et al. studied the effectiveness of three novel modified surface flow constructed wetlands (CW1: Brick rubble, lignite, and *Lemna minor* L.; CW2: Brick rubble and lignite; CW3: *Lemna minor* L.) in treating sugar factory wastewater. The results suggested that CW1 exhibited high Chao1, Shannon, and Simpson indices. The denitrifying bacterial class Rhodobacteriaceae was found to be the most abundant in CW1. This finding supported that CW1 enhances the performance of water filtration in constructed wetlands.

Xing et al. analyzed vegetation changes and their drivers in the Changbai Mountain alpine tundra. The results revealed a decline in typical alpine plants and an increase in herbaceous plants. Species richness and diversity showed an upward trend across various elevations, with a notable shift toward herbaceous dominance. The study found that soil nutrients, rather than climate, were the primary drivers of short-term vegetation changes.

We think that all accepted articles in this Research Topic will provide new knowledge on microbial processes in soil-vadose zone-groundwater habitats.
